# Molybdenum Disulfide‐Based Catalysts in Organic Synthesis: State of the Art, Open Issues, and Future Perspectives

**DOI:** 10.1002/smll.202406697

**Published:** 2024-10-20

**Authors:** Marc Morant‐Giner, Giuseppe Gentile, Maurizio Prato, Giacomo Filippini

**Affiliations:** ^1^ Instituto de Ciencia Molecular (ICMol) Universitat de València C/Catedrático José Beltrán 2 Paterna 46980 Spain; ^2^ Department of Chemical and Pharmaceutical Sciences INSTM UdR Trieste University of Trieste Via Licio Giorgieri 1 Trieste 34127 Italy; ^3^ Center for the Cooperative Research in Biomaterials (CIC BiomaGUNE) Basque Research and Technology Alliance (BRTA) Paseo de Miramón 194 Donostia‐San Sebastián 20014 Spain; ^4^ Basque Foundation for Science Ikerbasque Plaza Euskadi 5 Bilbao 48013 Spain

**Keywords:** heterogenous catalysis, molybdenum disulfide, organic chemistry

## Abstract

In the field of heterogeneous organic catalysis, molybdenum disulfide (MoS_2_) is gaining increasing attention as a catalytically active material due to its low toxicity, earth abundance, and affordability. Interestingly, the catalytic properties of this metal‐based material can be improved by several strategies. In this Perspective, through the analysis of some explicative examples, the main approaches used to prepare highly efficient MoS_2_‐based catalysts in relevant organic reactions are summarized and critically discussed, namely: i) increment of the specific surface area, ii) generation of the metallic 1T phase, iii) introduction of vacancies, iv) preparation of nanostructured hybrids/composites, v) doping with transition metal ions, and vi) partial oxidation of MoS_2_. Finally, emerging trends in MoS_2_‐based materials catalysis leading to a richer organic synthesis are presented.

## Graphical abstract

Within this Perspective, the main approaches used to prepare highly efficient MoS_2_‐based catalysts in relevant organic transformations are summarized and critically discussed, namely: 1) increment of the specific surface area, 2) generation of the metallic 1T phase, 3) introduction of vacancies, 4) preparation of nanostructured hybrids/composites, 5) doping with transition metal ions, and 6) partial oxidation of MoS_2_.

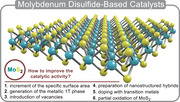

## 1 Introduction

**Figure 1:**
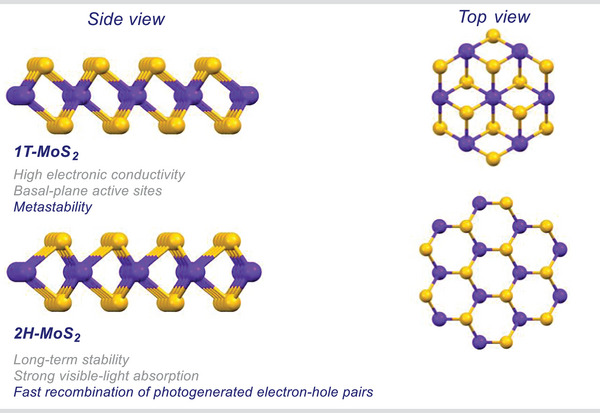
Different structural models of 1T and 2H polytypes in MoS_2_ monolayer. 2H monolayer is also referred to as 1H‐MoS_2_. Mo: Blue, S: Yellow.

The discovery of one‐atom‐thick graphene started a restless search for alternative 2D materials.^[^ ^1^ ^]^ Among such materials are layered transition metal dichalcogenides (TMDCs), a family of compounds with the chemical formula *MX*_2_, where *M* stands for a transition metal (usually belonging to groups IV–VII) and *X* refers to a chalcogen (like S, Se, or Te). The bulk structure of these materials is composed of *X*–*M*–*X* sandwiches that are held together by van der Waals interactions.^[^ ^2^ ^]^ As these interlayer interactions are much weaker than the in‐plane covalent bonding, individual *X*–*M*–*X* slabs can be isolated by dry and wet exfoliation methods.^[^ ^3^ ^]^ TMDCs exist predominantly as 1T, 2H, and 3R polytypes, where the alphanumeric code refers to the number of *X*–*M*–*X* layers per unit cell plus the structural symmetry (*T* = tetragonal, *H* = hexagonal, and *R* = rhombohedral).^[^ ^4^ ^]^ Molybdenum disulfide (MoS_2_) is an archetypal example of TMDC (**Figure** **1**).

While 1T‐MoS_2_ is a synthetic polytype with metallic character,^[^ ^5^ ^]^ 2H‐ and 3R‐MoS_2_ can be found in nature as mineral molybdenite and exhibit semiconducting properties.^[^ ^6^ ^]^ Interestingly, the downsizing of 2H‐MoS_2_ from bulk to monolayer happens alongside an indirect‐to‐direct bandgap transition.^[^ ^7^ ^]^ MoS_2_ holds great potential in heterogeneous catalysis both as a support and as an active component.^[^ ^8^ ^]^ As a matter of fact, bulk 2H‐MoS_2_ is a moderately toxic, Earth‐abundant, and therefore inexpensive material. Moreover, the exfoliation of this polytype represents an efficient way to obtain large‐area layers. Depending on whether an alkali‐containing intercalating agent (such as *n*‐butyllithium,^[^ ^9^ ^]^ sodium naphthalenide,^[^ ^10^ ^]^ or sodium‐potassium alloy)^[^ ^11^ ^]^ is used or not during the exfoliation process, these layers may contain a high proportion of 1T phase or be purely 2H. In this context, it is worth mentioning that liquid phase exfoliation methods are the most commonly used because they allow for the production of good quality exfoliated MoS_2_ in a simple and scalable manner. For example, Garrido and colleagues have recently reported the simultaneous exfoliation and functionalization of bulk 2H‐MoS_2_ with a tetrapyridyl porphyrin.^[^ ^12^ ^]^ In general, the exfoliation strategy not only permits the modification of the pristine electronic properties but also endows MoS_2_ with high specific surface area (SSA), rendering it more sensitive to the local chemical environment, which is desirable for boosting its catalytic efficiency. Another interesting possibility involves exploiting the chemistry of coordinatively unsaturated (CUS) Mo atoms (Lewis acid sites) located at sulfur vacancies (SVs) as well as at layer edges.^[^ ^13^ ^]^ From a catalytic point of view, it is worth mentioning that 1T and 2H polytypes have pros and cons. For instance, 1T‐MoS_2_ possesses high electronic conductivity and basal‐plane active sites but is metastable.^[^ ^14^ ^]^ On the contrary, 2H‐MoS_2_ shows long‐term stability and strongly absorbs light in the visible region,^[^ ^15^ ^]^ but suffers from fast recombination of photogenerated electron–hole pairs.^[^ ^16^ ^]^ Further research frontiers involve the doping of MoS_2_ with heteroatoms (such as oxygen)^[^ ^17^ ^]^ or transition metal ions (e.g., Co, Ni, and Cu, among others)^[^ ^18^ ^]^ and the possibility of interfacing MoS_2_ with another material to prepare hybrids/composites,^[^ ^19^ ^]^ thus combining their catalytic behaviors.

Despite the achievement of some successful applications over recent years, the use of MoS_2_ as catalyst/co‐catalyst for organic transformations is still in its infancy. Among other 2D materials, MoS_2_ possesses interesting properties that are still not fully exploited.^[^ ^20^ ^]^ Thus, this Perspective aims to describe, through the analysis of some explicative examples, the primary strategies employed for enhancing the catalytic performance of MoS_2_ in classical organic reactions. Finally, by means of the conclusion, future possibilities within this intriguing research field will be discussed.

## 2 Increasing the Specific Surface Area

The activity of a heterogenous catalyst is highly dependent on its SSA,^[^ ^21^ ^]^ as well as on the adsorption energy of the reactants on the solid surface.^[^ ^22^ ^]^ According to the Sabatier principle, the interaction between the catalyst surface and the reactants should be neither too strong nor too weak, in such a way that only a near‐zero value of free energy of adsorption would ensure a salient catalyst performance.^[^ ^23^ ^]^ In order to maximize the potential of the catalyst, MoS_2_ forms showing high surface‐area‐to‐volume ratios are strongly preferred over bulk counterparts, although the latter has also proven to efficiently catalyse several organic transformations, such as nitroarene reduction,^[^ ^24^ ^]^ isobutane dehydrogenation,^[^ ^25^ ^]^ transamidation reactions,^[^ ^26^ ^]^ and the condensation between indoles and benzaldehydes.^[^ ^19^, ^27^ ^]^ The reason behind this is that an increment of the SSA results in a larger number of catalytically active sites (generally CUS Mo atoms) ready for interacting with certain species present in the reaction medium. In relation with bulk structure, we demonstrated that the density of available Lewis acid catalytic sites present in commercially available 2H‐MoS_2_ increases after a straightforward thermal‐vacuum treatment.^[^ ^27^ ^]^ This process served to suitably desorb the adsorbates (such as water molecules) from the MoS_2_ surface. However, due to the intrinsic difficulties of using bulk 2H‐MoS_2_ (e.g., low surface area), the Design of Experiments (DoE) method was necessary to accelerate the optimization of the catalytic protocol. Unfortunately, after one catalytic cycle, the catalyst was partly poisoned. Although certain MoS_2_ nanoarchitectures with considerably large SSA, such as nanoflowers^[^ ^28^ ^]^ and nanosheets,^[^ ^29^ ^]^ were reported as catalysts in organic chemistry, their final catalytic activities were attributed to other effects (see Sections 3 and 4). In addition, spherical amorphous MoS_2_ particles, with an average diameter of ≈600 nm and a SSA of 32.63 m^2^ g^−1^, were employed for the reduction of 4‐nitrophenol (4‐NP) to 4‐aminophenol (4‐AP).^[^ ^30^ ^]^

**Figure 2:**
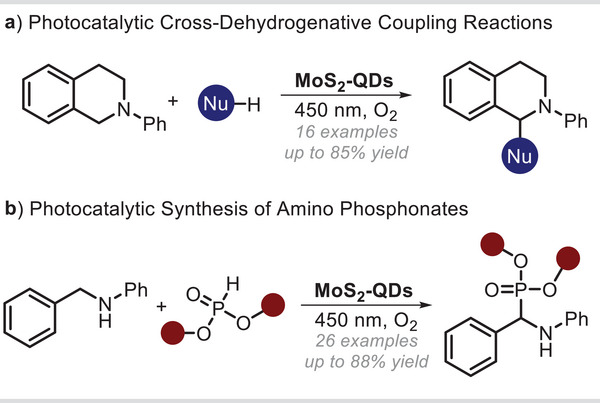
MoS_2_‐based QDs as heterogeneous photoredox catalysts. a) Photocatalytic cross‐dehydrogenative coupling reactions. b) Photocatalytic synthesis of α‐amino phosphonate derivatives. A: electron acceptor; D: electron donor; Nu: Nucleophile.

Nevertheless, for this particular reaction, the reusability of the amorphous catalyst was not supported by experiments. Within the nanoparticle range, semiconductive quantum dots (QDs) possess the largest surface‐to‐volume ratio and excellent photophysical features.^[^ ^31^ ^]^ Further, controlling the dimensions of QDs appears to be an effective strategy to modulate the optical properties of these materials.^[^ ^32^ ^]^ For instance, reducing the dimensions of QDs size shifts both absorption and emission toward higher energy in agreement with that expected by quantum confinement effect.^[^ ^33^ ^]^ Besides, quantum confinement effect also causes the bandgap to increase when the dimensions of the nanostructure are lower or comparable with the excitonic Bohr radius.^[^ ^34^ ^]^ Consequently, reducing the size of QDs can be an effective strategy to increase the reduction potential, simultaneously allowing for a faster transfer of electrons from the surface.^[^ ^35^ ^]^ In this scenario, De and co‐workers described a bottom–up approach for the production of MoS_2_ QDs and their application as heterogeneous photocatalysts to drive the functionalization of tetrahydroisoquinoline derivatives (**Figure** **2**a).^[^ ^36^ ^]^ Specifically, these MoS_2_‐based nanoparticles, with a size of 6–8 nm are capable of absorbing light up to 500 nm. Upon visible‐light radiation, the resultant photogenerated holes oxidase the starting materials (namely tetrahydroisoquinolines), thus initiating their derivatization. The studied C─C and C─P bond forming reactions generate good yields (up to 85%) over 16 reported examples, delivering a high number of densely functionalized organic products under mild operative conditions. The authors observed that the yield of coupling products was influenced by the substitution at the nitrogen center of indole (nucleophile), with yields decreasing as the bulkiness of the substituent increased. Importantly, the reusability of the photocatalyst was also demonstrated for the model transformation for (at least) four cycles, without any significant detriment in the product yield. It is worth noting that these readily available nanomaterials are emerging as green and economical alternatives to classical transition metal‐based photocatalysts based of ruthenium and iridium complexes.^[^ ^37^ ^]^ Following a similar approach, the same research group has recently produced and used organo‐soluble MoS_2_‐based QDs as heterogeneous recyclable photoredox catalysts to drive the synthesis of relevant α‐amino phosphonates, which are bio‐isosteres of amino acids and recurring moieties in numerous pharmaceutical drugs (Figure 2b).^[^ ^38^ ^]^ In particular, the authors have demonstrated that these photocatalytic systems, upon light absorption and in the presence of molecular oxygen, are capable of effectively producing reactive iminium ion intermediates from suitable *N*‐phenyl benzylamines. These electrophilic intermediates eventually react with different phosphite derivatives, yielding the desired products (28 examples) with good to excellent yields. The scope of this transformation demonstrated high tolerance for various substituents and substitution patterns on *N*‐phenyl benzylamine. Moderate to good yields were observed when neither side of *N*‐phenyl benzylamine was substituted. Electron‐donating substituents enhanced yields, possibly by stabilizing the in situ formed iminium ion, while electron‐withdrawing groups on the benzylic moiety reduced yields due to iminium ion instability. Substituting electron‐donating and electron‐withdrawing groups on opposite sides of the substrate resulted in high yields, but placing electron‐withdrawing groups on both sides significantly lowered yields, highlighting the role of aromatic substitution. Notably, the authors successfully synthesized tryptamine derivatives, which are bioactive compounds, with excellent yields. Moreover, the processes could be scaled up to a 6 mmol scale without significant loss of yield. In contrast to certain archetypical II–VI QDs (such as CdS), 2H‐MoS_2_ photocatalysts offer some advantages: i) low toxicity;^[^ ^39^ ^]^ ii) wider spectral absorption (cut‐off wavelength ranging from near‐infrared to visible spectral) and more efficient solar spectrum utilization;^[^ ^23b^ ^]^ iii) photocorrosion resistance;^[^ ^39^, ^40^ ^]^ and iv) the possibility of improving the separation of the photoinduced charges in combination with the metallic 1T phase, without using other materials.^[^ ^41^ ^]^

Lastly, it is worth mentioning that Brunauer–Emmett–Teller (BET) analysis is the most widely recognized technique to determine the SSA of solid samples.

## 3 Preparing the 1T Polytype

Although 1T‐MoS_2_ provides more electrochemically active sites along the basal plane than 2H‐MoS_2_,^[^ ^42^ ^]^ the metastable nature of the former represents a serious issue when using and recycling the catalyst. However, some mixed‐phase MoS_2_ forms (i.e., with the coexistence of 1T and 2H phases) have been demonstrated to be stable and catalytically active. Interestingly, this type of material displays unique physicochemical features that result in novel catalytic behaviors. In this scenario, chemically exfoliated MoS_2_ nanosheets, with a percentage of 1T polytype of ≈76%, were utilized as catalysts for the reduction of 4‐NP to 4‐AP.^[^ ^29^ ^]^ It has been suggested that the enhanced catalytic performance is related to the concurrence of metallic 1T sites, which would facilitate the electron transfer from the reductant to substrate molecules and strain in the chemically exfoliated material. The reusability of the catalyst was only tested for the reduction of ferricyanide. Unfortunately, some important aspects remained unclear, including the chemical nature of the material after its use and the optimal 1T/2H ratio needed to achieve the highest catalytic performance. Remarkably, Girish et al. showed that metal–semiconductor heterojunctions in mixed‐phase MoS_2_ nanosheets, with a percentage of 1T phase of ≈72%, could greatly increase the productivity in the photocatalytic oxidative coupling of benzylamines when compared to exfoliated 2H‐MoS_2_.^[^ ^41^ ^]^

The photocatalytic strategy enables the efficient preparation of imines from benzylamines containing both electron‐withdrawing and electron‐donating groups, without a clear difference in the conversion rates depending on the nature of the substituents. Notably, the catalyst also facilitates the oxidation of 1,2,3,4‐tetrahydroquinoline to quinoline, demonstrating its versatility in promoting diverse oxidative transformations. Indeed, this group proves that heterojunctions facilitate the separation of the photogenerated electron/hole, therefore favoring the charge transfer process between the excited catalyst and the reacting molecules. The semiconducting 2H polytype is responsible for generating electron–hole pairs under light irradiation, and the photogenerated electrons are transferred to metallic 1T phase. What's more, in this regard, mixed‐phase MoS_2_ nanoflowers, with a percentage of 1T polytype of ≈64%, are used to drive the hydrodesulfurization (HDS) of dibenzothiophene (DBT),^[^ ^28^ ^]^ which is a widely used process in the industry for eliminating sulfur containing compounds from fuels. In this case, the high catalytic activity arises from a stable combination of 1T/2H phases with structural defects (see next section). At this point, it is important to remark that the chemical exfoliation of bulk 2H‐MoS_2_ based on the use of *n*‐butyllithium (*n*‐BuLi) not only induces the polytype transition to the metallic phase but also generates structural defects that provide additional active sites. In the papers by Girish^[^ ^41^ ^]^ and Cao,^[^ ^28^ ^]^ the real 1T phase percentage remaining after using the catalyst is unclear because of an incomplete deconvolution of Mo X‐ray photoelectron spectroscopy (XPS) peaks. Li group achieved to increase the content of 1T phase in 1T/2H mixed nanoflowers up to ≈80% by using oxalic acid and studied their potential in HDS reaction, but without proposing a mechanism.^[^ ^43^ ^]^ As pointed out by research results, high 1T percentages are generally required for catalytic enhancements. Nevertheless, it would be interesting to find out whether there is a limit 1T/2H ratio above which the catalytic activity of MoS_2_ for a particular organic reaction starts to decrease.

Usually, the quantification of 1T and 2H percentages is performed by XPS.

## 4 Introducing Defects: S and Mo Vacancies

Another strategy to boost the catalytic activity of MoS_2_ is to play with the density of sulfur and molybdenum vacancies (SVs and MoVs, respectively), which can be exploited as active sites for some organic chemical transformations. Owing to their lower energy formation, SVs are more employed than MoVs. Despite the variety of ad hoc methods for generating SVs in MoS_2_, it is important to note that the formation of vacancies is intrinsic to the exfoliation process. Thus, the presence of these structural defects as well as the increment of SSA may contribute to the final catalytic performance. The hydrazine‐assisted exfoliation of 2H‐MoS_2_ yields nanosheets, with a concentration of SVs of 5.44 × 10^17^ g^−1^, capable of effectively promoting the hydrodeoxygenation (HDO) of 4‐methylphenol.^[^ ^44^ ^]^ Nevertheless, the recyclability of this heterogeneous catalytic material is not tested. Concerning specific post‐exfoliation methods, efficient MoS_2_ catalysts toward the reduction of 4‐NP to 4‐AP are prepared by treating colloidal suspensions with hydrazine.^[^ ^45^ ^]^ Curiously, the best results are obtained with intermediate concentrations of SVs. The enhanced catalytic performance is attributed to the role of SVs as local electron donors, which can ease the electron transfer in the involved redox reactions. In this case, the catalyst is immobilized on melamine foam and recycled several times with substantial retention of the activity, but not characterized after its use. Interestingly, vacancies can also be created during or after the synthetic procedure. On the one hand, defective mixed‐phase MoS_2_ nanoflowers containing SVs and MoVs (S/Mo atomic ratio = 1.86) are directly obtained by using a solvothermal approach.^[^ ^28^ ^]^ On the other hand, Zhang et al. demonstrated that, after hydrothermal synthesis, the calcination of 2H‐MoS_2_ at 450 °C under N_2_ induced the formation of both SVs and MoVs. This material was used to catalyze the one‐pot reductive amination of nitro compounds with aldehydes to yield secondary amines.^[^ ^46^ ^]^ In view of experimental results and density functional theory (DFT) calculations, SVs may act as active sites for the hydrogenation of intermediate imines to yield the final secondary amines. The main limitation of this catalyst was that, if exposed to air for a few days, it was deactivated because surface oxygen species possibly occupied MoS_2_ defects. The reaction scope included 11 entries, covering both a series of functionalized nitroarenes and benzaldehyde derivatives. Both electron‐donating and electron‐withdrawing substituents were successfully employed, yielding secondary amines in good to excellent yields. As anticipated, nitroarenes with electron‐donating groups produced higher yields compared to those with electron‐withdrawing groups.

Usually, the detection of vacancies is based on XPS and, to a lesser extent, on extended X‐ray absorption fine structure (EXAFS). If the S/Mo atomic ratio determined by XPS is significantly lower than 2 (MoS*_x_*, *x* < 2), the presence of SVs is confirmed. Alternatively, if the coordination numbers (CNs) of Mo─S and Mo─Mo shells extracted from Mo K‐edge EXAFS spectrum are both smaller than the theoretical values (CN_Mo─S_ and CN_Mo─Mo_ = 6), the MoS_2_‐based catalyst contains SVs and MoVs, respectively.^[^ ^46^ ^]^

## 5 Producing Nanostructured Hybrids/Nanocomposites

**Figure 3:**
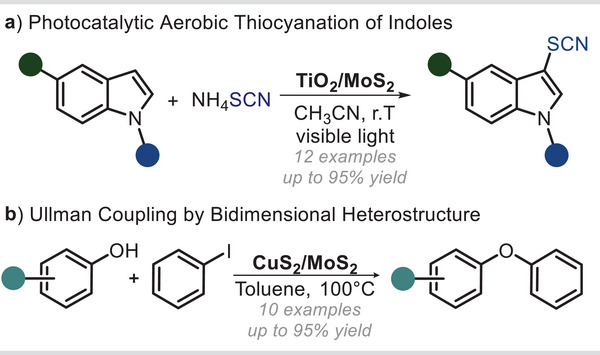
MoS_2_‐based composites materials. a) Photocatalytic aerobic thiocyanation of substituted indoles. b) Ullman type coupling of phenols with iodobenzene.

The preparation of nanostructured hybrids/composites, obtained by combining MoS_2_ with other suitable materials, is emerging as an effective approach to produce catalysts with unforeseen characteristics.^[^ ^47^ ^]^ As highlighted previously, the native semiconductor behavior of 2H‐MoS_2_ has made it an appealing material for crafting visible‐light‐responsive photocatalysts.^[^ ^48^ ^]^ In addition, combining MoS_2_ with a different material could result in the obtaining of heterojunctions (p−n) or Z‐schemes, which are two of the most modern and elegant approaches for achieving significant enhancement of photocatalytic performance.^[^ ^48b^ ^]^ These strategies have proven to be successful in developing MoS_2_‐photocatalyzed transformations, including hydrogen evolution, CO_2_ reduction, degradation of organic pollutants, and the oxidation/reduction of small organic molecules.^[^ ^48^, ^49^ ^]^ However, this approach remains relatively unexplored for the synthesis of fine chemicals. In this context, Wang et al. provided an example by developing a photocatalytic system based on TiO_2_ nanoparticles modified with MoS_2_ layers.^[^ ^47a^ ^]^ Specifically, this material was produced through a bottom–up approach starting from TiCl_4_, sodium molybdate, and thioacetamide as the sulfur source. The obtained composite material showed high photocatalytic activity toward the aerobic thiocyanation of indoles (**Figure** **3**a).

Importantly, the presence of MoS_2_ within the catalytic structure, capable of absorbing light within the visible region, meant that the use of highly energetic and harmful UV‐light could be avoided. This aspect not only reduces the overall cost of the process but also minimizes the risk to operators’ health. The reaction scope was successfully extended to 16 entries, showing good tolerance for various functional groups, such as chloro, bromo, methoxyl, methyl, and phenyl. However, substrates containing electron‐withdrawing groups remained unreactive, even with extended reaction times. To highlight the practicality of this protocol, a scale‐up reaction on a 10 mmol scale was conducted. Notably, the transformation yielded the desired product in 83% after a prolonged reaction time. Last, the photocatalyst showed stability under the operative conditions and was recycled up to eight times. As mentioned before, a common drawback that often impedes the direct application of bulk MoS_2_ is its low surface area, which makes active sites scarcely available to interact with the desired substrate.^[^ ^46^, ^50^ ^]^ Besides, bulk MoS_2_ generally suffers from inhomogeneous dispersion in common organic solvents. This aspect tends to reduce the interaction between the reagents and the material's surface and, in turn, the productivity of the overall process. To resolve this issue, some successful applications have relied on the assembly of MoS_2_ with other 2D materials to increase the surface area, and thus, the activity of the resulting catalyst. For instance, Xie and colleagues recently described a bottom–up procedure for the synthesis of a composite material made of MoS_2_ and CuS_2_. This novel 2D hetero‐nanostructure is capable of effectively driving Ullman coupling reactions (Figure 3b).^[^ ^19a^ ^]^ Specifically, the authors started from the exfoliation of bulk 2H‐MoS_2_ powder in liquid phase to increase the surface area of the material. Afterward, the nanosheets were refluxed with a source of copper (II) cations, namely Cu(acetate)_2_. Electrostatic interactions caused the copper ions to be drawn to the surface of the larger nanosheets. Then, these cations could react with S^2−^ to form Cu_2_S directly on the surface of MoS_2_ nanosheets. The overall process was aided by the strong polarity of the solvent, namely *N*,*N*‐dimethylformamide (DMF), which was optimal for both dispersing the nanosheets and stabilizing the metal cations. Finally, the material was collected by centrifugation and rinsed with ethanol. The material obtained drove the coupling reaction of substituted phenols with iodobenzene in toluene to obtain the corresponding ethers. The scope of this reaction, although limited to iodobenzene and simple substituted phenols, indicated that the protocol was suitable for the preparation of aromatic ethers with both electron‐withdrawing and electron‐donating groups on the aromatic ring. Interestingly, neither bulk MoS_2_ nor the physical mixture of Cu_2_S and MoS_2_ could drive the reaction to an appreciable yield.

**Figure 4:**
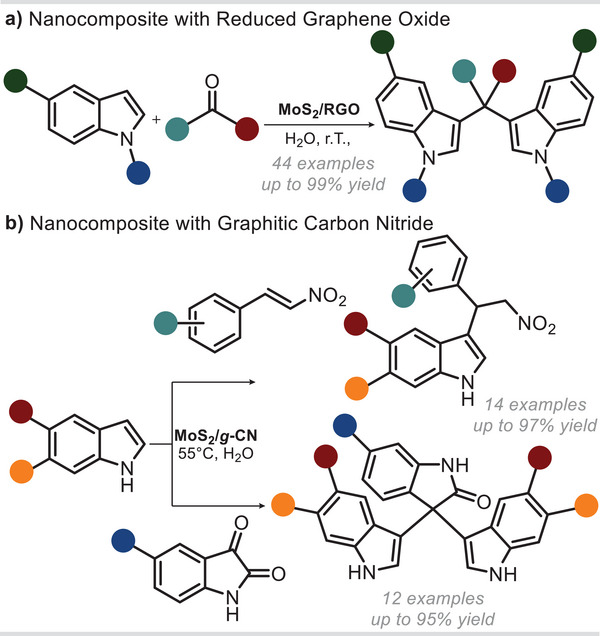
MoS_2_‐based nanocomposites materials. a) Catalytic alkylation of substituted indoles with aldehydes and ketones. b) Catalytic alkylation of substituted indoles with nitro alkenes and isatine derivatives.

The intrinsic Lewis acidity of TMDCs can also be exploited to catalyze valuable organic reactions. To this purpose, Krishnan and collaborators prepared nanocomposites by coupling MoS_2_ with 2D carbon nanomaterials, namely reduced graphene oxide (RGO) and graphitic carbon nitride (g‐CN). In particular, these materials were able to catalyze the C‐3 functionalization of indoles with carbonyl compounds (**Figure** **4**).^[^ ^19^, ^51^ ^]^ MoS_2_/RGO nanocomposite was produced through a single‐step procedure.^[^ ^19b^ ^]^ Specifically, graphene oxide (GO) was mixed with Na_2_MoO_4_·2H_2_O and L‐cysteine and thermally treated in an autoclave. The material showed a superior catalytic activity in driving the C‐3 functionalization of indoles with aldehydes and ketones when compared with the deriving components (Figure 4a). The authors attributed the enhanced catalytic activity to a higher hydrophilicity and higher surface area of the composite. The scope of this reaction proved to be quite broad, encompassing 44 examples, including some of pharmaceutical relevance. Specifically, electron‐donating substituents on the indole ring promoted the reaction, while electron‐withdrawing atoms within the indole ring (e.g., 7‐azaindole) hindered it. Interestingly, electron‐withdrawing substituents (such as F, Cl, Br, CN, and NO₂) on the indole ring had no significant effect on reactivity in the synthesis of bisindolylmethanes. Further, the presence of electron‐donating or electron‐withdrawing groups on the aldehyde portion, unlike on the indole ring, did not noticeably impact reactivity. The reaction also proceeded just as effectively with various alkyl groups on the indole nitrogen atom.

The same research group followed a comparable approach to produce a nanoheterostructure with g‐CN and MoS_2_ meant for C‐3 functionalization of indoles with nitroalkenes and isatine derivatives (Figure 4b).^[^ ^51^ ^]^ In this example, the g‐CN and MoS_2_ nanosheets were prepared separately and assembled by solvent dispersion. The obtained heterogeneous catalyst (MoS_2_/g‐CN) could effectively promote the reaction of indoles with carbonyl compounds and nitroalkenes. This could be explained by considering the better wettability of the composite, with respect to pristine MoS_2_, paired with a rougher texture and higher surface area. The authors claimed that the excellent catalytic activity of this composite could be attributed to the synergistic effects of the two components, allowing for the formation of Lewis acid–base non‐bonding adducts. A broad substrate scope was demonstrated by varying the substituents on indoles and isatins. The reactivity of isatin remained consistent with indoles containing either electron‐withdrawing or electron‐donating groups, except for 5‐nitroindole. In addition, the authors showcased the catalytic methodology's potential by conducting a gram‐scale synthesis of a precursor of substituted tryptamine, a phenyl derivative of serotonin.

However, it is worth pointing out that, if we confront the production rate normalized by the surface area of MoS_2_/*g*‐CN versus that of the bulk MoS_2_ used in the catalytic comparison, it follows that the productivity value was lower for the composite than for the bare MoS_2_ (0.114 mmol m^−2^ h^−1^ vs 0.131 m^−2^ h^−1^, respectively). Consequently, it is unclear whether the higher catalytic activity could be attributed to the presence of g‐CN or just to the increased surface area. Regarding the preparation of nanostructured hybrids/composites, it is worth mentioning that most of the reference works suffered from i) deficient/incomplete characterization of the constituent components of the catalytic system or ii) no characterization of the recycled catalyst.

Last, from an experimental point of view, the constituent components of the catalytic systems were generally identified by characterization techniques as diverse as powder X‐ray diffraction (PXRD), Raman spectroscopy, Fourier‐transform infrared (FTIR) spectroscopy, and energy‐dispersive X‐ray spectroscopy (EDXS) elemental mapping.

## 6 Doping with Transition Metals

**Figure 5:**
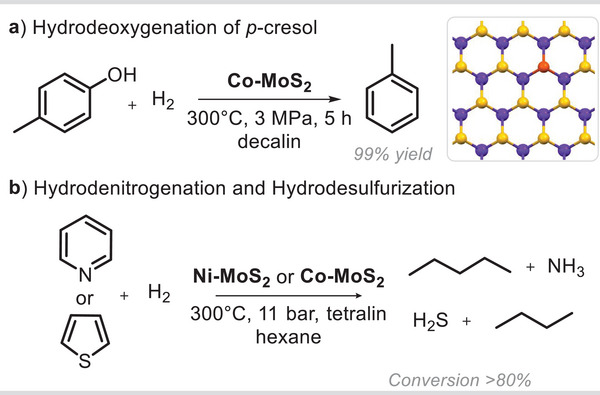
MoS_2_‐based metal‐doped materials. a) Selective hydrodeoxygenation of p‐cresol to toluene. b) Hydrodenitrogenation and hydrodesulfurization of heteroarenes under mild operative conditions. Inset: atomic model of doped‐MoS_2_ single layer. S: yellow, Mo: blue, Co or Ni: orange.

The doping of MoS_2_ with certain transition metal ions generates new effective active centers, thus improving the catalytic efficiency for specific organic transformations. For instance, Co‐doped MoS_2_ proved to be selective toward the conversion of 4‐methylphenol to toluene (**Figure** **5**a), an example of HDO reaction.^[^ ^52^ ^]^ In this example, Co‐doped monolayers, with a 1.8% weight loading of Co, were prepared by a time‐consuming procedure (more than 4 days) consisting of several steps, where the final step involved the annealing of the material at 300 °C under H_2_. During this process, Co atoms incorporated into the basal plane promoted the creation of SVs in proximal sites. Also in relation to HDO, Zhang et al. achieved an enhancement of the catalytic activity based on the generation of surface acidic sites (responsible for the C─O bond cleavage) by H_2_O_2_ etching.^[^ ^53^ ^]^

In this case, the experimental observations revealed that the HDO activity of Co‐doped MoS_2_ was dependent on the amount of Brønsted acid and Lewis acid sites. Unfortunately, the material was not characterized after its use. Another reaction catalyzed by Co‐ and Ni‐doped MoS_2_ was the reduction of 4‐NP to 4‐AP.^[^ ^18^, ^54^ ^]^ According to some authors, Co‐substituted sites not only accelerated the rate of electron transfer reactions thanks to the Co^2+^/Co^3+^ redox couple but also helped to stabilize the metallic (and therefore conductive) 1T phase in mixed‐phase MoS_2_.^[^ ^54^ ^]^ Nevertheless, in the two aforementioned articles,^[^ ^18^, ^54^ ^]^ the retention of 1T polytype after recycling was not experimentally proved. Of interest was the study of the catalytic potential of *M*‐doped MoS_2_ (*M* = Mn, Fe, Co, Ni, Cu, or Zn) toward the 4‐NP reduction reaction reported by Liu group.^[^ ^18b^ ^]^ The prominent activity of Ni‐doped MoS_2_, with a 5.9% weight loading of Ni, was attributed to the expansion of the interlayer spacing (0.94 nm), which provided more active sites and the weakening of adsorbent–adsorbate interactions, facilitating the desorption of surface hydrogen species from the catalyst. As the content of molybdenum oxides was not comparable between the different samples, the question of whether this parameter may affect the catalytic performance remains open. Another missing aspect is the interlayer distance observed in the rest of *M*‐doped MoS_2_ systems. If compared to bulk, Co and Ni‐doped monolayers are also suitable catalysts for the HDS of thiophene and the hydrodenitrogenation (HDN) of pyridine, respectively (Figure 5b).^[^ ^18a^ ^]^ For a better understanding of the effect of *M* ^III^/*M* ^II^ ratio on the catalytic properties, Co‐ and Ni‐doped samples should have been prepared using cobalt(II) and nickel(II) acetylacetonate, respectively, where the transition metals were in the same oxidation state.

To calculate the concentration of dopants in MoS_2_ catalysts, the most common characterization techniques are XPS and inductively coupled plasma‐optical emission spectrometry (ICP‐OES). The spatial distribution of these single‐atom dopants can also be visualized by EDXS elemental mapping.

## 7 Preparing Partially Oxidized MoS_2_

The partial oxidation of MoS_2_ can result in a boosting of the catalytic performance. Partially oxidized MoS_2_ nanorods (with a theoretical formula MoS_2−_ *_x_* O*_x_*, where *x* is close to 1) obtained from the incomplete sulfurization of α‐MoO_3_ analogues were effective on the HDS of DBT.^[^ ^55^ ^]^ Notwithstanding, no information about the possibility of recycling the catalyst or about how molybdenum oxides contribute to the final catalytic activity was reported. Oxygen‐implanted 2D MoS_2_ (with a 3.7% atomic percentage of O extracted from EDXS analysis) prepared by hydrothermal synthesis was able to catalyze the reduction of nitroarenes to the corresponding arylamines.^[^ ^56^ ^]^ The high catalytic performance was attributed to a cooperative effect between the active Mo^IV^O*_x_* structure and its surrounding MoS_2_ skeleton. The activity of this material toward the one‐pot quinoline synthesis starting from basic nitroarenes and aliphatic alcohols was later reported.^[^ ^17^ ^]^ Unfortunately, in neither of these two studies^[^ ^17^, ^56^ ^]^ was the catalyst characterized after its use. The question of whether this catalytic system can be further optimized by playing with the Mo^IV^O*_x_*/MoS_2_ ratio remains unanswered.

As in the case of dopant atoms, the spatial distribution of oxygen can also be visualized by EDXS elemental mapping.

## 8 Conclusions and Perspectives

In recent years, the growing attention to sustainable chemical processes has stimulated the development of novel heterogeneous MoS_2_‐based catalytic systems. In fact, these low‐toxic and inexpensive materials are emerging as excellent candidates due to their tailorable surface and excellent physicochemical features. Therefore, these MoS_2_‐containing materials have been exploited as effective catalytic and photocatalytic platforms for several organic transformations.

Specifically, the examples discussed within this Perspective underline the recent progress toward the preparation and application of MoS_2_‐based catalysts in polar and light‐driven chemical reactions of synthetical importance. Although promising results have been obtained so far, a number of unsolved challenges and opportunities remain unaddressed.

We expect that in the coming years, research on this topic will evolve to the design of i) new chiral heterogeneous MoS_2_‐based catalysts for enantioselective organic reactions (for example, functionalizing MoS_2_ surface with chiral organic molecules), ii) novel dual catalytic synthetic strategies involving coupling MoS_2_ catalysis with a second catalytic route to exploit possible synergy in more challenging chemical conversions or multiple products, and iii) tailored QD‐based photocatalysts capable of exploiting low energetic sources of photons, such as green (*λ* = 525 nm) or yellow (*λ* = 580 nm) lamps, to drive valuable organic transformations.

Moreover, if rapid progress in the field is to be made, all research on heterogeneous catalysis should provide the following details: i) the yield of the studied reaction (with and without the catalyst) expressed as an average of a number of measurements, ii) a clear explanation on the nature (SVs, metallic 1T sites, etc.) and localization of the catalytic sites, iii) the proposed reaction mechanism, iv) recycling studies, and v) an accurate characterization of the catalyst before and after its use. In relation to the first point, it is important to note that the vast majority of papers provide reaction yield values without standard deviation, which suggests that some of the reported percentages have not been calculated as the average of several measurements; and therefore, could suffer from poor reproducibility. Another aspect to consider is that an incomplete characterization may give the impression that the catalytic performance of a given material is due to a specific effect when in fact there are several effects at play. For instance, for nanosheets obtained by chemical exfoliation with *n*‐BuLi, the observed catalytic activity can be attributed to the presence of metallic 1T sites, but the generation of additional new vacancies as well as the increment of SSA with respect to bulk MoS_2_ should also be considered. The use of DoE, a powerful data analytics tool, can significantly reduce experimental work by identifying and optimizing the critical parameters affecting a particular catalyzed reaction, as has been recently demonstrated.^[^ ^27^ ^]^

To conclude, we foresee that future investigations on MoS_2_‐based catalytic materials will help to resolve the related present‐day challenges in organic synthesis in academia and industry.

## Conflict of Interest

The authors declare no conflict of interest.

## References

[1] K. S. Novoselov , A. Mishchenko , A. Carvalho , A. H. Castro Neto , Science 2016, 353, aac9439. 27471306 10.1126/science.aac9439

[2] a) J. A. Wilson , A. D. Yoffe , Adv. Phys. 1969, 18, 193.

[3] Q. Zhang , L. Mei , X. Cao , Y. Tang , Z. Zeng , J. Mater. Chem. A. 2020, 8, 15417.

[4] M. Chhowalla , H. S. Shin , G. Eda , L.‐J. Li , K. P. Loh , H. Zhang , Nat. Chem. 2013, 5, 263. 23511414 10.1038/nchem.1589

[5] I. Song , C. Park , H. C. Choi , RSC Adv. 2015, 5, 7495.

[6] a) A. D. Marinov , L. Bravo Priegue , A. R. Shah , T. S. Miller , C. A. Howard , G. Hinds , P. R. Shearing , P. L. Cullen , D. J. L. Brett , ACS Nano 2023, 17, 5163. 36926849 10.1021/acsnano.2c08913 PMC10062033

[7] A. Kuc , N. Zibouche , T. Heine , Phys. Rev. B: Condens. Matter Mater. Phys. 2011, 83, 245213.

[8] P. Chandra , A. Mohammad , B. Tripathi , T. Yoon , FlatChem 2022, 34, 100395.

[9] P. Joensen , R. F. Frindt , S. R. Morrison , Mater. Res. Bull. 1986, 21, 457.

[10] J. Zheng , H. Zhang , S. Dong , Y. Liu , C. T. Nai , H. S. Shin , H. Y. Jeong , B. Liu , K. P. Loh , Nat. Commun. 2014, 5, 2995. 24384979 10.1038/ncomms3995

[11] E. Er , H.‐L. Hou , A. Criado , J. Langer , M. Möller , N. Erk , L. M. Liz‐Marzán , M. Prato , Chem. Mater. 2019, 31, 5725.

[12] M. Garrido , A. Criado , M. Prato , Nanoscale 2024, 16, 13525. 38946392 10.1039/d4nr01802h

[13] S. Rangarajan , M. Mavrikakis , ACS Catal. 2017, 7, 501.

[14] Z. Lei , J. Zhan , L. Tang , Y. Zhang , Y. Wang , Adv. Energy Mater. 2018, 8, 1703482.

[15] G. Uliana , G. Valdrè , J. Appl. Cryst. 2023, 56, 611. 37284254 10.1107/S1600576723002571 PMC10241059

[16] a) W. Cui , S. Xu , B. Yan , Z. Guo , Q. Xu , B. G. Sumpter , J. Huang , S. Yin , H. Zhao , Y. Wang , Adv. Electron. Mater. 2017, 3, 1700024.

[17] C. Zhang , Z. Gao , P. Ren , J. Lu , Z. Huang , K. Su , S. Zhang , J. Mu , F. Wang , Green Chem. 2022, 24, 1704.

[18] a) K. Guo , Y. Ding , Z. Yu , Appl. Catal., B 2018, 239, 433.

[19] a) X. Sun , H. Deng , W. Zhu , Z. Yu , C. Wu , Y. Xie , Angew. Chem., Int. Ed. 2016, 55, 1704. 10.1002/anie.201508571 26669284

[20] C. Rosso , G. Filippini , A. Criado , M. Melchionna , P. Fornasiero , M. Prato , ACS Nano 2021, 15, 3621. 33715354 10.1021/acsnano.1c00627 PMC8041367

[21] S. Özkar , Appl. Surf. Sci. 2009, 256, 1272.

[22] D. Voiry , J. Yang , M. Chhowalla , Adv. Mater. 2016, 28, 6197. 26867809 10.1002/adma.201505597

[23] a) J. K. Nørskov , T. Bligaard , J. Rossmeisl , C. H. Christensen , Nat. Chem. 2009, 1, 37. 21378799 10.1038/nchem.121

[24] L. Huang , P. Luo , M. Xiong , R. Chen , Y. Wang , W. Xing , J. Huang , Chin. J. Chem. 2013, 31, 987.

[25] E. Cheng , L. McCullough , H. Noh , O. Farha , J. Hupp , J. Notestein , Ind. Eng. Chem. Res. 2019, 59, 1113.

[26] F. Zhang , L. Li , J. Ma , H. Gong , Sci. Rep. 2019, 9, 2536. 30796297 10.1038/s41598-019-39210-5 PMC6385372

[27] G. Gentile , M. Morant‐Giner , L. Cardo , M. Melchionna , P. Fornasiero , M. Prato , G. Filippini , ChemSusChem 2023, 16, 202300831. 10.1002/cssc.202300831 37486452

[28] H. Cao , Z. Bai , Y. Li , Z. Xiao , X. Zhang , G. Li , ACS Sustainable Chem. Eng. 2020, 8, 7343.

[29] L. Guardia , J. I. Paredes , J. M. Munuera , S. Villar‐Rodil , M. Ayán‐Varela , A. Martínez‐Alonso , J. M. Tascón , ACS Appl. Mater. 2014, 6, 21702. 10.1021/am506922q 25405770

[30] N. Saha , A. Sarkar , A. B. Ghosh , A. K. Dutta , G. R. Bhadu , P. Paul , B. Adhikary , RSC. Adv. 2015, 5, 88848. 10.1039/d3ra90043f PMC10231424 37266510

[31] Y. Guo , J. Li , Mater. Sci. Eng. C. 2020, 109, 110511. 10.1016/j.msec.2019.110511 32228919

[32] M. Khan , ChemistrySelect 2019, 4, 2116.

[33] S. Mukherjee , R. Maiti , A. K. Katiyar , S. Das , S. K. Ray , Sci. Rep. 2016, 6, 29016. 27357596 10.1038/srep29016 PMC4928078

[34] S. Golovynskyi , M. Bosi , L. Seravalli , B. Li , Surf. Interfaces 2021, 23, 100909.

[35] L. Chen , S. L. Hsieh , C. H. Kuo , S. Hsieh , W. H. Chen , C. W. Chen , C. D. Dong , RSC Adv. 2020, 10, 31794. 35518143 10.1039/d0ra04512h PMC9056492

[36] K. Jaiswal , Y. R. Girish , P. Behera , M. De , ACS Org. Inorg. Au 2022, 2, 205. 36855472 10.1021/acsorginorgau.1c00040 PMC9955124

[37] M. H. Shaw , J. Twilton , D. W. C. MacMillan , J. Org. Chem. 2016, 81, 6898. 27477076 10.1021/acs.joc.6b01449 PMC4994065

[38] A. Kayal , M. De , ChemCatChem 2024, 16, 202400264.

[39] H. Ullah , Z. Haneef , A. Ahmad , I. S. Butler , R. N. Dara , Z. Rehman , Inorg. Chem. Commun. 2023, 153, 110775.

[40] Y. Yang , Y. Zhang , Z. Fang , L. Zhang , Z. Zheng , Z. Wang , W. Feng , S. Weng , S. Zhang , P. Liu , ACS Appl. Mater. Interfaces 2017, 9, 6950. 28145695 10.1021/acsami.6b09873

[41] Y. R. Girish , R. Biswas , M. De , Chem.‐Eur. J. 2018, 24, 13871. 29932251 10.1002/chem.201802468

[42] Z. W. Seh , J. Kibsgaard , C. F. Dickens , I. B. Chorkendorff , J. K. Nørskov , T. F. Jaramillo , Science 2017, 355, eaad4998. 28082532 10.1126/science.aad4998

[43] Z. Baia , L. Wang , H. Cao , X. Zhang , G. Li , Fuel 2022, 322, 124252.

[44] G. Liu , H. Ma , I. Teixeira , Z. Sun , Q. Xia , X. Hong , S. C. E. Tsang , Chem.‐Eur. J. 2016, 22, 2910. 26757235 10.1002/chem.201504009

[45] S. García‐Dalí , J. I. Paredes , B. Caridad , S. Villar‐Rodil , M. Díaz‐González , C. Fernández‐Sánchez , A. Adawy , A. Martínez‐Alonso , J. M. D. Tascón , Appl. Mater. Today 2020, 20, 100678.

[46] Y. Zhang , Y. Gao , S. Yao , S. Li , H. Asakura , K. Teramura , H. Wang , D. Ma , ACS Catal. 2019, 9, 7967.

[47] a) L. Wang , C. Wang , W. Liu , Q. Chen , M. He , Tetrahedron Lett. 2016, 57, 1771.

[48] a) Z. Li , X. Meng , Z. Zhang , J. Photochem. Photobiol., C Photochem. Rev. 2018, 35, 39.

[49] a) P. P. Singh , S. Sinha , G. Pandey , V. Srivastava , RSC Adv. 2022, 12, 29826. 36321108 10.1039/d2ra05695j PMC9578401

[50] a) L. Li , Z. Qin , L. Ries , S. Hong , T. Michel , J. Yang , C. Salameh , M. Bechelany , P. Miele , D. Kaplan , M. Chhowalla , D. Voiry , ACS Nano 2019, 13, 6824. 31136708 10.1021/acsnano.9b01583

[51] A. Bahuguna , A. Kumar , S. Kumar , T. Chhabra , V. Krishnan , ChemCatChem 2018, 10, 3121.

[52] G. Liu , A. W. Robertson , M. M.‐J. Li , W. C. Kuo , M. T. Darby , M. H. Muhieddine , Y.‐C. Lin , K. Suenaga , M. Stamatakis , J. H. Warne , Nat. Chem. 2017, 9, 810. 28754945 10.1038/nchem.2740

[53] Y. Zhang , T. Liu , Q. Xia , H. Jia , X. Hong , G. Liu , J. Phys. Chem. Lett. 2021, 12, 5668. 34114828 10.1021/acs.jpclett.1c01201

[54] a) C. Nethravathi , J. Prabhu , S. Lakshmipriya , M. Rajamathi , ACS Omega 2017, 2, 5891. 31457843 10.1021/acsomega.7b00848 PMC6644400

[55] M. A. Albiter , R. Huirache‐Acuña , F. Paraguay‐Delgado , J. L. Rico , G. Alonso‐Núñez , Nanotechnology 2006, 17, 3473. 19661592 10.1088/0957-4484/17/14/020

[56] C. Zhang , Z. Zhang , X. Wang , M. Li , J. Lu , R. Si , F. Wang , Appl. Cat A Gen. 2016, 525, 85.

